# The amygdala: securing pleasure and avoiding pain

**DOI:** 10.3389/fnbeh.2013.00190

**Published:** 2013-12-06

**Authors:** Anushka B. P. Fernando, Jennifer E. Murray, Amy L. Milton

**Affiliations:** Department of Psychology, Behavioural and Clinical Neuroscience Institute, University of CambridgeCambridge, UK

**Keywords:** amygdala, appetitive and aversive conditioning, memory, pavlovian, corticostriatal pathway

## Abstract

The amygdala has traditionally been associated with fear, mediating the impact of negative emotions on memory. However, this view does not fully encapsulate the function of the amygdala, nor the impact that processing in this structure has on the motivational limbic corticostriatal circuitry of which it is an important structure. Here we discuss the interactions between different amygdala nuclei with cortical and striatal regions involved in motivation; interconnections and parallel circuitries that have become increasingly understood in recent years. We review the evidence that the amygdala stores memories that allow initially motivationally neutral stimuli to become associated through pavlovian conditioning with motivationally relevant outcomes which, importantly, can be either appetitive (e.g. food) or aversive (e.g. electric shock). We also consider how different psychological processes supported by the amygdala such as conditioned reinforcement and punishment, conditioned motivation and suppression, and conditioned approach and avoidance behavior, are not only psychologically but also neurobiologically dissociable, being mediated by distinct yet overlapping neural circuits within the limbic corticostriatal circuitry. Clearly the role of the amygdala goes beyond encoding aversive stimuli to also encode the appetitive, requiring an appreciation of the amygdala's mediation of both appetitive and fearful behavior through diverse psychological processes.

## Introduction

The amygdala, an important structure within the limbic forebrain, interacts with both the cortex and the striatum to influence motivated behavior. The amygdala itself has long been associated with emotional processing, particularly the emotion of fear. Monkeys with amygdala damage are typically tame and apparently fearless (Klüver and Bucy, 1939); rats with amygdala lesions do not show fear behavior such as pavlovian *conditioned freezing* or *fear-potentiated startle* (see Table 1 for definitions of specialized terms, indicated in italicised text) (Ledoux et al., 1990; Kim et al., 1993; Campeau and Davis, 1995; Maren et al., 1996) and humans with calcification of the amygdala seen in Urbach-Wiethe disease also show deficits in remembering emotionally arousing material (Siebert et al., 2003). However, it has been appreciated since the time of Weiskrantz (1956) that the amygdala is not limited to representing fearful stimuli, but it is also required for the performance of appetitive tasks in which individuals work for rewards such as sex or drugs of abuse (Cador et al., 1989; Everitt et al., 1989; Burns et al., 1993).

**Table 1:** **Definitions of specialized psychological and behavioral terms**.

**Term**	**Definition**
Acquisition of a new instrumental response for conditioned reinforcement (ANR)	Behavioral procedure used to study appetitive *conditioned reinforcement*. Animals are presented with a CS (e.g. a tone) that is paired with an appetitive reinforcer (e.g. food) during pavlovian conditioning. The manipulandum for a new instrumental response (e.g. a lever) is then presented to the animal. Responding on one of the levers produces the CS; the other control lever has no consequence. If the CS is a conditioned reinforcer then it should support the acquisition of a new response even when the primary reinforcer (i.e. the food) is no longer presented.
Active avoidance	Behavioral procedure in which animals are trained to make an instrumental response in the presence of a discrete CS or context in order to avoid the presentation of an aversive reinforcer (e.g. an electric footshock). This procedure can be contrasted with “passive avoidance,” in which animals are required to remain where they are in order to avoid electric shock.
Autoshaping	Behavioral procedure used to study *conditioned approach* or appetitive *conditioned direction.* Animals are trained to associate a readily-localizable CS (e.g. a light-lever combination) with the presentation of an appetitive reinforcer through pavlovian conditioning. During training, the CS comes to elicit approach responses that may be directed towards the CS itself (termed *sign-tracking*) or towards the location in which the reinforcer is delivered (termed *goal-tracking*). Often a control CS, not associated with a reinforcer, is included in the procedure, and animals are considered to have acquired the association when they approach more during presentation of the reinforcer-associated CS than during the control CS.
Avoidance	An instrumentally conditioned action that prevents an aversive reinforcer from occurring.
Conditioned approach	The psychological process by which a CS acquires reinforcing properties that promote approach towards it; often the CS will also elicit responses that are appropriate to the reinforcer (e.g. a rat will lick a CS associated with a liquid reinforcer).
Conditioned direction	Our suggested term to encompass both *conditioned approach* and *avoidance*, since both processes depend upon the same neural circuitry and the CS performs the same directing function in both processes.
Conditioned freezing	The rodent-specific fear response of the cessation of all movement, except for respiration, in the presence of a fear-eliciting stimulus.
Conditioned inhibitor	A CS that suppresses or reduces the number or size of conditioned response that would be elicited by presentation of another CS. Conditioned inhibition is usually measured through “summation” tests (in which the excitor and inhibitor CS are presented simultaneously, and levels of responding compared to the presentation of the excitor CS alone) and in “retardation of acquisition” tests, in which the conditioned inhibitor is associated with another outcome, which produces delayed learning compared to control CSs that have not previously been trained as conditioned inhibitors.
Conditioned motivation	The psychological process by which a pavlovian CS affects levels of instrumental responding. This term is often used synonymously with *pavlovian-instrumental transfer*, but we suggest that this term should be used more generally to refer to both *pavlovian-instrumental transfer* and *conditioned suppression*.
Conditioned reinforcement	The psychological process by which a pavlovian CS acquires conditioned, or secondary, reinforcing properties that allow it to support instrumental responding (e.g. as measured using the *ANR* procedure). This term is often used to refer to the appetitive conditioned reinforcing properties of a CS, but we suggest that it should also refer to *conditioned punishment*, as both processes depend upon the same neural circuitry, and the CS is presented following the response, either as a positive reinforcer in appetitive conditioned reinforcement or as a negative reinforcer in conditioned punishment.
Conditioned punishment	The psychological process by which a pavlovian CS acquires conditioned, or secondary, aversive reinforcing properties (i.e. the stimulus becomes feared) such that it promotes avoidance of a particular instrumental response. We suggest that as conditioned punishment depends upon the same neural circuitry as appetitive conditioned reinforcement, that it is more parsimonious to term conditioned punishment “aversive conditioned reinforcement.”
CS-specific properties of an association	The sensory-specific properties of a pavlovian CS—for example, a specific frequency of tone or light—associated to a specific outcome or pavlovian US. The association of the sensory-specific properties of a pavlovian CS is hypothesized to depend upon the basolateral amygdala.
Conditioned stimulus	In pavlovian conditioning, a previously motivationally neutral stimulus that is associated with an unconditioned stimulus (reward or reinforcer).
Conditioned suppression	The capacity of an aversive pavlovian CS to suppress ongoing instrumental responding.
Devaluation	The reduction in value of a reinforcer by either associating the reinforcer with an unpleasant outcome (e.g. gastric malaise induced by lithium chloride injection for food reinforcers) or by reducing the motivation for the reinforcer (e.g. by allowing free access to the reinforcer prior to testing, as in sensory-specific satiety procedures).
Extinction	The process by which the response to a previously learned association (pavlovian or instrumental) is reduced. Procedurally, pavlovian extinction occurs through presentation of the CS without the US, and instrumental extinction occurs by omitting reinforcement following the previously-reinforced response. Importantly, extinction is not “unlearning” of the previously learned association, but instead reflects the formation of a new, inhibitory “CS-no US” or “action-no outcome” memory that inhibits the original memory in a context-specific manner.
Fear-potentiated startle	The increase in startle response produced by a stimulus (e.g. a loud noise) when it is presented in the presence of a fear-eliciting stimulus or an anxiogenic environment.
General properties of an association	The generalized motivational properties of a pavlovian association—for example, the association between a pavlovian CS and an appetitive motivational outcome, though not necessarily the association between the CS and a specific outcome. The encoding of the generalized properties of an association is hypothesized to depend upon the central nucleus of the amygdala.
General PIT	In *pavlovian-instrumental transfer*, the process by which any appetitive CS can enhance instrumental responding for an appetitive reinforcer (cf. *specific PIT*).
Goal-directed	In instrumental conditioning, the association by which an action that produces a particular outcome (or goal state) is represented. Responses are elicited depending upon the representation of the outcome, so that if the outcome is *devalued* then the action will not be elicited.
Goal-tracking	*Conditioned approach* towards the location in which the reinforcer is delivered when a pavlovian CS, associated with an appetitive reinforcer, is presented.
Instrumental conditioning	A type of learning in which the outcome is dependent upon the behavior of the individual. Learning can occur through positive reinforcement (increasing the number of responses that produce an appetitive reinforcer), *negative reinforcement* (increasing the number of responses that allow the individual to avoid an aversive reinforcer) or punishment (decreasing the number of responses that produce an aversive reinforcer).
Negative reinforcement	A type of *instrumental conditioning* procedure in which a particular behavior is increased in frequency due to the avoidance of an aversive outcome.
Pavlovian conditioning	A type of learning in which a previously motivationally neutral stimulus is paired in space and time with a motivationally relevant unconditioned stimulus. The behavior of the individual does not affect the contingency between the presentation of the two stimuli.
Pavlovian-instrumental transfer (PIT)	The behavioral procedure with which appetitive *conditioned motivation* can be assessed. Animals are trained separately on an instrumental association and a pavlovian association for the same reinforcer. Responses made in the presence of the pavlovian CS can be taken as a direct test of conditioned motivational properties of the CS (without the CS acting to induce retrieval of the instrumental action representation).
Pearce-Hall model of learning	A model of pavlovian conditioning which predicts that individuals pay greater attention to events that are surprising, which facilitates learning.
Prediction error	During a behavioral experience, the mismatch between what is expected based on prior experience and what actually occurs. Prediction error is hypothesized to drive learning in many theories, including the Rescorla–Wagner model of learning. Neurobiologically, prediction error correlates with levels of midbrain dopamine signaling.
Rescorla–Wagner model of learning	A model of pavlovian conditioning in which individuals are hypothesized to learn about the association between pavlovian CSs and USs based on *prediction error* (i.e. learning occurs when there is a mismatch between the prediction of, and the actual delivery, of the US). Changes in the prediction of likelihood (“associative strength,” Δ*V*_*x*_) are determined by the salience of the CS (α), the ease of learning about the CS (β) and the degree of learning about the US that has already occurred (i.e. the difference between the total amount of learning that could theoretically occur about the CS, λ, and what has been learned so far, V_*tot*_). This is represented by the Rescorla–Wagner equation, Δ*V*_*x*_ = αβ(λ – *V*_*tot*_).
Safety signal	A pavlovian CS which, when presented, indicates that an aversive reinforcer will not be delivered.
Second-order schedule	Behavioral procedure often used to measure the conditioned reinforcing properties of a CS. Under a second-order schedule, animals are trained to associate an instrumental response with both an appetitive reinforcer and a pavlovian CS. During training, the response requirements are increased such that a certain number of responses will produce the CS, and a certain number of CSs, or responses within a certain period of time, will produce the reinforcer.
Sign-tracking	*Conditioned approach* towards the location of a pavlovian CS when the CS, associated with an appetitive reinforcer, is presented.
Specific PIT	In *pavlovian-instrumental transfer*, the process by which a CS associated with a specific appetitive reinforcer can enhance instrumental responding for the same reinforcer.
Stimulus-response	In instrumental conditioning, the association by which a pavlovian CS elicits a response, which is independent of the representation of the outcome. If responding is habitual (stimulus-response) then it will be maintained even if the outcome of the action has been *devalued*. Stimulus-response learning typically occurs following overtraining, or training in which the contingency between the response and the outcome is degraded (e.g. interval schedules).
Stimulus saliency	The capacity of a stimulus to direct attention. This could be due to the physical attributes of the stimulus (e.g. intensity) but is often also related to the motivational relevance of a CS.
Unconditioned stimulus	In pavlovian conditioning, a stimulus that is motivationally relevant to the individual (e.g. food, water, sex).

Each term is italicized in the text at its first appearance.

A compelling hypothesis (Ono et al., 1995; Robbins and Everitt, 2002; Balleine and Killcross, 2006) is that the amygdala functions as a memory storage device, encoding the association of initially motivationally neutral environmental stimuli (“cues”) with motivationally relevant outcomes in a pavlovian manner. Therefore, the amygdala can be thought of as associating these pavlovian cues—“*conditioned stimuli*” (CSs)—with motivationally relevant “*unconditioned stimuli*” (USs) such as food, sex, or danger. The amygdala is considered a good candidate neural structure for storing these pavlovian memories, as it receives highly processed sensory information (that constitutes a CS) from the sensory cortices, and this converges on the amygdala with other “raw” sensory inputs, such as visceral or gustatory afferents that can represent the outcome or the US (Li et al., 1996; McDonald et al., 1999). This convergence of CS and US information occurs at the level of individual neurons: though neurons within the amygdala are responsive to both unimodal and multimodal stimuli without any prior experience (Bordi and Ledoux, 1994), the number of stimulus-responsive neurons in the amygdala increases following pavlovian training (Uwano et al., 1995), suggesting that synaptic plasticity is occurring at the neuronal level in the amygdala following associative learning. Importantly, electrophysiological studies show that the amygdala responds to CSs that have been associated with either appetitive or aversive outcomes (Paton et al., 2006; Tye and Janak, 2007), supporting the view that the amygdala stores both appetitive and aversive pavlovian “CS-US” memories.

In a complex, naturalistic environment, animals can enhance their chances of obtaining pleasure (i.e. motivationally relevant rewards like food and sex) and avoiding pain and danger by using pavlovian environmental cues to guide behavior. For example, for a rat, associating the smell of cat urine with a fearful motivational state may give the rat an evolutionary selective advantage, namely that it is more likely to avoid environments in which cat urine is present, and so reduce the risk of predation. Representing the emotional and affective value of pavlovian CSs is therefore important from an evolutionary perspective; however, the amygdala does not act alone in this function, but rather interacts with a larger corticostriatal motivational circuit (Cador et al., 1989). The connections between the amygdala and the ventral striatum provide a major route by which the amygdala can affect motivated behavior. The ventral striatum has been hypothesized to represent potential actions within the behavioral repertoire (Liljeholm and O'Doherty, 2012), from which actions can be selected for specific motivated behaviors (for example, food-seeking or mate-seeking behaviors). The amygdala allows pavlovian CSs to influence the selection of actions within the behavioral repertoire; so, for instance, in the presence of a receptive female, a male rat is more likely to engage in courtship behavior than food-seeking. However, the amygdala is not a unitary structure, but is divided into several subnuclei, with major divisions including the central (CeN), basal and lateral (considered here together as the basolateral amygdala, or BLA) and the corticomedial nuclei. These subnuclei are hypothesized to have different functions in representing pavlovian CSs. For the purposes of the current discussion, the most relevant divisions are the CeN and the BLA.

The BLA and CeN are heavily interconnected, forming a circuit through which sensory information about pavlovian CSs can be integrated to produce coordinated emotional responses, including neuroendocrine, autonomic and behavioral responses. However, until relatively recently there has been disagreement regarding the connections between the BLA and CeN; the views essentially being divided between “serial processing” models, in which the BLA controlled the responses of the CeN, which itself formed the “output nucleus” of the amygdala (Ledoux, 2000; Maren, 2001) and “parallel processing” models, in which the BLA and CeN both receive sensory inputs, and can influence different aspects of motivated behavior in parallel (Cardinal et al., 2002a; Everitt et al., 2003; Balleine and Killcross, 2006; Wilensky et al., 2006). The “parallel processing” model has become increasingly accepted in recent years, being supported by behavioral, neuroanatomical and electrophysiological evidence. Thus, we begin by considering the requirement for the BLA and CeN in behavior measured with different psychological tasks that help to dissociate the functions of these nuclei, for appetitive and aversive tasks, and those tasks that depend upon interactions between the appetitive and aversive motivational systems. We then review how the neuroanatomical connections of the CeN and BLA, and the response properties of amygdala neurons, support the parallel model, for both appetitive and aversive CSs.

## The amygdala stores both appetitive and aversive pavlovian memories

According to the parallel processing model of amygdala function (Cardinal et al., 2002a; Everitt et al., 2003; Balleine and Killcross, 2006; Wilensky et al., 2006), the BLA and CeN both store pavlovian CS-US memories, but the two nuclei encode different types of information about the CS, and consequently influence behavior in different ways. The BLA, which receives highly processed information from the sensory cortices, is hypothesized to represent the specific affective value of a CS and to influence *goal-directed instrumental* behavior; supporting a function similar to other areas of cortex. By contrast, the CeN is hypothesized to represent more generalized associations based upon the motivational valence of the reinforcer (Swanson and Petrovich, 1998; Cardinal et al., 2002a) and to influence *stimulus-response* behavior, similar to regions of the dorsal striatum. Though a pavlovian CS is capable of supporting both *CS-specific associations* and *general associations*, these processes can be separated using specific behavioral procedures developed by learning theorists (see Cardinal et al., 2002a; Milton and Everitt, 2010, for review) and these will be discussed in more detail below. As an illustrative example, however, consider work by Corbit and Balleine (2005) investigating *conditioned motivation*. In this study, rats were trained on an instrumental task to press two different levers for different food outcomes (pellets and sucrose). Subsequently, the same animals were then trained separately on a pavlovian task to associate different auditory stimuli with the pellets and sucrose. The animals were tested for “*pavlovian-instrumental transfer*” (PIT) by allowing them to press the two levers while the auditory stimuli were presented. In non-lesioned control animals, presentation of the auditory CS associated with sucrose led to a marked increase in pressing the lever associated with sucrose (known as “*specific PIT*”) and a smaller increase in pressing of the lever associated with pellets (known as “*general PIT*”). Similar effects were observed with the pellet-associated CS; an increase in responding on both levers, with a greater enhancement in responding on the pellet-associated lever. In amygdala-lesioned animals, behavior was markedly and differentially affected by the subdivision of the amygdala that was lesioned. While animals that received BLA lesions still showed an enhancement in lever pressing in the presence of the auditory stimuli, they did not show the specific enhancement shown by controls (e.g. an increase in pressing the sucrose-associated lever in the presence of the sucrose-CS). Animals with CeN lesions, by contrast, continued to show specific enhancement, but did not show the general increase in lever pressing shown by controls. Thus, by using carefully designed behavioral procedures, it is possible to dissociate the functions of the BLA and CeN.

A number of different psychological processes have been identified by which a pavlovian CS can influence instrumental behavior. In the past, there has been a tendency to separate these processes based upon whether the association is between a CS and an appetitive or aversive reinforcer. When appetitive reinforcers are used, these processes are termed pavlovian *conditioned approach* (approach toward a pavlovian CS; measured through a procedure known as “*autoshaping*”), conditioned motivation (the enhancement of instrumental responding by the presentation of a pavlovian CS; measured through PIT as described above) and *conditioned reinforcement* (the phenomenon by which a pavlovian CS acquires reinforcing properties in its own right; measured using, for example, a procedure known as the *acquisition of a new instrumental response for conditioned reinforcement*). Other procedures that measure *conditioned suppression* (a reduction in instrumental responding in the presence of a pavlovian CS previously associated with an aversive outcome) and *avoidance* (e.g. of a pavlovian CS associated with an aversive outcome), have been developed with aversive stimuli. We suggest that these appetitive and aversive processes are recruiting the same neural circuitry, and it may therefore be more parsimonious to group together appetitively and aversively motivated behavior based on common psychological processes. We suggest that PIT and conditioned suppression are measuring the capacity of a pavlovian CS to enhance (if it is appetitive) or reduce (if it is aversive) previously trained instrumental behavior, and therefore these psychological processes could be grouped together under the term “*conditioned motivation*.” Conditioned approach and avoidance essentially describe directional behavior to the pavlovian CS (toward or away) and thus we suggest that these terms might be united under the term “*conditioned direction*.” Finally, pavlovian CSs can become reinforcing in their own right through association with the motivationally relevant US, whether the US is appetitive (so the CS is a “conditioned reinforcer”) or aversive (so the CS is a “conditioned punisher”). Again, we suggest that these are essentially the same psychological process, which could be considered as “conditioned reinforcement” (encompassing both appetitive *conditioned reinforcement*, and aversive conditioned reinforcement, respectively). These processes differentially depend upon CS-specific and general associations and, therefore, differentially upon the BLA and CeN.

### The BLA is required for sensory-specific associations

#### The BLA is required for appetitive and aversive conditioned reinforcement

An appetitive conditioned reinforcer is defined as a pavlovian CS, initially of motivationally neutral valence, which acquires reinforcing properties through its association with the US (Mackintosh, 1974). The often-cited human example of an appetitive conditioned reinforcer is money, which has no primary reinforcing value, but because of its previous association with motivationally relevant outcomes (e.g. food) it acquires conditioned affective and reinforcing properties. Money, like all appetitive conditioned reinforcers, will support delays to primary reinforcement (i.e. individuals will work for money over an extended time period in order to save for a particularly expensive purchase) and it will support the acquisition of new instrumental behaviors (i.e. if offered a sufficiently large monetary reward, most individuals would be motivated to acquire a new skill). Appetitive conditioned reinforcement is extremely persistent, and strongly resistant to *extinction* (Di Ciano and Everitt, 2004); individuals will continue responding for an appetitive conditioned reinforcer long after it was last predictive of the US, and individuals will also continue responding even when the US has itself been *devalued* (Parkinson et al., 2005).

Appetitive conditioned reinforcement depends upon the BLA, as has been shown in several behavioral tasks that allow conditioned reinforcement to be measured in isolation. The appetitive conditioned reinforcing properties of CSs, associated with appetitive reinforcers, can be assessed using procedures such as *second-order schedules* (see Everitt and Robbins, 2000, for review), in which long delays to primary reinforcement are supported by the presentation of conditioned reinforcers; learning of this task depends upon the BLA (Burns et al., 1999). Appetitive conditioned reinforcement can also be measured using “the acquisition of a new instrumental response with conditioned reinforcement” procedure (“ANR”; Hyde, 1976), in which individuals are trained first to make an instrumental response for the primary reinforcer and the pavlovian CS, and in a second phase of training are tested on their ability to acquire a novel instrumental response for presentations of the CS alone. Pre-training excitotoxic lesions of the BLA impair the acquisition of responding under second-order schedules for CSs associated with different primary reinforcers, including sex (Cador et al., 1989; Everitt et al., 1989), cocaine (Whitelaw et al., 1996; Arroyo et al., 1998; Goddard and Leri, 2006), and food (Hatfield et al., 1996), and likewise ANR is also impaired by excitotoxic lesions of the BLA in rats (Burns et al., 1993) and monkeys (Parkinson et al., 2001). Reversible lesions, induced by inactivation of the BLA with the glutamate (AMPA) receptor antagonist CNQX, also prevents discriminated responding for a previously amphetamine-associated CS during a test of conditioned reinforcement (Hitchcott and Phillips, 1997). This deficit is also observed with mice lacking AMPA receptors within the BLA (*gria1* knockouts), which are impaired on tests of conditioned reinforcement (Mead and Stephens, 2003b). Specifically, BLA lesions appear to prevent the CS from influencing the selection of the appropriate instrumental actions, such that animals without a functioning BLA are unable to use the CS to guide their instrumental action selection, whether that is an increase in lever pressing for a stimulus associated with reward in second-order schedules or ANR, or avoidance of an instrumental response that produces an aversive outcome.

The aversive conditioned reinforcing properties of CSs can be assessed using procedures such as “conditioned punishment” (Killcross et al., 1997a,b), in which an instrumental response is associated with the probabilistic presentation of an aversive event such as an electric footshock paired with a CS. As for appetitive conditioned reinforcement, animals with BLA lesions are also impaired on aversive conditioned reinforcement (Killcross et al., 1997b); BLA-lesioned animals do not bias responding away from a lever associated with electric footshock and an aversive CS, though they still show reduced overall responding on the lever and on a control lever not paired with shock, i.e. intact conditioned suppression (Killcross et al., 1997b; Purgert et al., 2012).

It is worth noting that recent work has begun to dissociate conditioned reinforcement into general and specific forms (see Burke et al., 2007, for details) with both forms being impaired by amygdala lesions. However, though amygdala lesions impaired performance on tasks measuring conditioned reinforcement (Burke et al., 2007), the lesions affected both the CeN and BLA, so on the basis of the evidence currently available it is not possible to attribute general and specific associations to specific amygdala subdivisions in the same manner as for other psychological processes (see below). Overall, the majority of evidence suggests that conditioned reinforcement depends on the BLA.

#### The BLA is required for specific forms of conditioned motivation

Studies of conditioned motivation are assessed using PIT procedures. There are two versions of PIT tasks—one that does not distinguish between the specific and general forms of conditioned motivation, and one that does. In the simplest form of the task, animals are trained separately on a pavlovian training phase, to associate a CS^+^ (e.g. a tone) with a reinforcer (e.g. sucrose) and an alternative CS^−^ (e.g. a clicker) with no reinforcement, and on instrumental training, where a response (e.g. lever pressing) produces the sucrose reinforcer. The animals are subsequently tested by allowing them to make the instrumental response (in the absence of the primary sucrose reinforcer) in the presence of the pavlovian CSs for the first time. The alternative version of the task is similar, but allows for the sensory-specific and generalized properties of CSs to be dissociated behaviorally (e.g. as described above for the study by Corbit and Balleine, 2005). The procedure for distinguishing between the sensory-specific and generalized properties of pavlovian CSs relies on associating responses and CSs with specific outcomes: for example, in the first stage of training, one response (e.g. lever pressing) may be associated with a specific outcome (e.g. sucrose) while another response (e.g. chain pulling) is associated with another outcome (e.g. sugar pellets); and in the second stage of training, a CS (e.g. a light) is associated with one outcome (sucrose) while a different CS (e.g. a tone) is associated with the other outcome (pellets). This leads to the formation of two instrumental associations (“lever pressing-sucrose” and “chain pulling-sugar pellets”) and two pavlovian associations (“light-sucrose” and “tone-pellets”). During testing, the animal is allowed to make the instrumental responses in the presence of the pavlovian CSs for the first time, in the absence of the primary reinforcer. Presentation of the CSs typically leads to an enhancement of instrumental responding, which is designated as “pavlovian-instrumental transfer” (PIT), or appetitive conditioned motivation. The presentation of the CSs can produce both “general” and “specific” effects on instrumental responding; the enhancement in instrumental responding produced by the CS associated with the same reinforcer (e.g. the effect of the light on lever pressing, or the tone on chain pulling) is termed “specific” conditioned motivation, and the enhancement in instrumental responding produced by the other CS (e.g. the effect of the light on chain pulling, or the tone on lever pressing) is termed “general” conditioned motivation. It is thought that specific conditioned motivation occurs because the presentation of the CS activates the stored CS-US memory, which in turn activates the response-reinforcer association; general conditioned motivation, by contrast, is thought to reflect a general excitation and facilitatory effect on behavior produced by an appetitive CS.

The BLA is required for specific conditioned motivation. Lesions of the BLA impair the performance of specific PIT (e.g. would impair the capacity of the light to potentiate lever pressing, or the tone to potentiate chain pulling in the example above) without affecting the generalized excitatory properties (e.g. the capacity of the light to potentiate chain pulling, or the tone to potentiate lever pressing) of the reinforcers in rats (Blundell et al., 2001; Corbit and Balleine, 2005) and the BLA is activated when human subjects are performing an outcome-specific PIT task (Prévost et al., 2012). By contrast, the BLA is not required for appetitive PIT (Hall et al., 2001; Holland and Gallagher, 2003) in versions of the task where a CS^+^ is reinforced and a CS^−^ is not reinforced; in this type of task, the potentiation of instrumental behavior can be supported by the general, non-CS-specific association, which is thought to depend upon the CeN rather than the BLA (see below).

#### The BLA is required for discriminative conditioned direction

Conditioned direction, particularly appetitive conditioned approach, can also be separated into CS-specific and general forms. Conditioned approach is produced by associating a pavlovian CS, usually with a distinct location for procedures that measure *sign-tracking* (i.e. a light, rather than an auditory stimulus like a tone, which is more difficult for animals to localize) with a reinforcer. Over time, presentation of the CS elicits either an approach response directed toward the CS—sometimes designated “sign-tracking”—or toward the location in which the reinforcer is delivered—“*goal-tracking*.” There are individual differences in the propensity to develop sign-tracking and goal-tracking responses, which appear to be correlated with dopaminergic signaling within the limbic corticostriatal circuitry (Flagel et al., 2007). As for PIT, conditioned approach procedures can be adapted to measure specific or general approach. In the general form of the procedure, animals are trained that a CS^+^ is predictive of reinforcer delivery, while a CS^−^ is not. In the specific form of the procedure, one CS (e.g. a solid light, in one location) is paired with delivery of a specific reinforcer (e.g. sucrose) while another CS (e.g. a flashing light, in a different location) is paired with an alternative reinforcer (e.g. pellets). For both types of procedure, animals are tested on their propensity to approach either the CSs or the location of reinforcer delivery in the absence of primary reinforcement.

Although lesions of the BLA have been shown to leave conditioned approach intact in a number of studies (Hatfield et al., 1996; Parkinson et al., 2000; Cardinal et al., 2002b), these studies all measured generalized approach; therefore, the lack of effect of BLA lesions on this form of conditioned approach is consistent with the hypothesis that the BLA is not required for generalized CS-US associations. The BLA does, however, appear to be necessary for pavlovian conditioned approach toward a specific CS (Ostlund and Balleine, 2008). Animals with BLA lesions show at test indiscriminate approach toward CSs that are predictive of reinforcement, including those for which the reinforcement contingency has been degraded (Ostlund and Balleine, 2008) and BLA lesions also reduce the final rates of responding in conditioned approach procedures (Chang et al., 2012b). One possible explanation for these effects suggests that there is a differential dependence on amygdala subnuclei at different stages of acquisition of conditioned approach. Using the general form of conditioned approach as an example, early in acquisition, animals typically approach both the reinforced CS^+^ and non-reinforced (control) CS^−^ indiscriminately, before reducing approach to the CS^−^ in the later stages of training. Thus, the early stage of approach training may depend upon the general affective properties of the CSs, while the later stages of training, when animals begin to discriminate between the CS^+^ and CS^−^, may be more dependent upon the sensory-specific properties of the CS^+^ (Chang et al., 2012b). Consistent with this, animals with BLA lesions would be predicted to acquire an indiscriminate approach response, but to be insensitive to devaluation, changes in temporal contiguity, or reinforcement contingency, which is consistent with experimental observations (Ostlund and Balleine, 2008).

Thus, a major function of the BLA can be considered as allowing a specific CS-US association to influence instrumental behavior (Cardinal et al., 2002a). This view accounts for the data reviewed above, and also for the finding that the acquisition of *active avoidance* responses in which an instrumental response is made in order to avoid a negative outcome (i.e. *negative reinforcement*) depends upon the BLA (Choi et al., 2010; Lázaro-Muñoz et al., 2010).

### The CeN is required for more generalized CS effects on responding, including general conditioned motivation and conditioned direction

In contrast to the BLA, the CeN is hypothesized to reflect more generalized, “excitatory” effects of pavlovian CSs on instrumental responding, and it may also support some simple stimulus-response learning (Cardinal et al., 2002a). It may also encode the “salience” of CSs, determining the amount of attention given to them: CeN lesions reduce orienting responses to a pavlovian CS paired with food, suggesting a reduction in attention to reinforcing stimuli (Gallagher et al., 1990); inactivation of the CeN releases exploratory behavior in the elevated plus maze, which is indicative of reduced attention to the anxiogenic effects of being in the open arms (Moreira et al., 2007); and lesions produce deficits in performance on a 3-choice serial reaction time task consistent with impairments in attentional processing (Holland et al., 2000).

Many studies of amygdala representations of pavlovian CSs have focused on demonstrating a double dissociation in the effects of lesions of the BLA and CeN. Lesions of the CeN neither impair appetitive conditioned reinforcement (Hatfield et al., 1996; Robledo et al., 1996) nor aversive conditioned reinforcement (Killcross et al., 1997b). However, the standard methodology for assessing conditioned reinforcement in isolation (e.g. using ANR) depends upon the sensory-specific properties of the pavlovian CS; therefore, if the hypothesized dissociation between the BLA (specific) and CeN (general) responses is correct then it would not be expected that CeN lesions would produce deficits on these tasks. Whether CeN lesions produce deficits on the versions of the task that allow general and specific conditioned reinforcement to be dissociated (Burke et al., 2007) remains to be investigated.

#### The CeN is required for general conditioned motivation

As described above, tasks assessing conditioned motivation readily allow for the separation of behavior supported by general and sensory-specific properties of the pavlovian CS. “General PIT,” in which presentations of a CS enhance responding for an affectively consistent, though different, outcome, is impaired by lesions of the CeN (Corbit and Balleine, 2005), as is PIT in which only a single response type is measured, which is likely supported by the general excitatory properties of the CS (Hall et al., 2001; Holland and Gallagher, 2003).

Conditioned suppression procedures measure aversive conditioned motivation with a similar experimental logic to PIT tasks. In conditioned suppression tasks, a CS^+^ is generally associated with an aversive reinforcer (e.g. an electric footshock) and the capacity of this CS to suppress instrumental responding (e.g. licking at a drinking spout for thirsty rats, or lever pressing for a sucrose reinforcer) is measured during the testing phase. Thus, in the same manner that an appetitive pavlovian CS can potentiate instrumental responding, an aversive pavlovian CS reduces instrumental responding at test. However, tests of conditioned suppression tend to use only a single aversive reinforcer (e.g. footshock) associated with a CS^+^, rather than having two aversive outcomes associated with different CSs; in this sense, conditioned suppression tasks are most comparable to tests of general PIT. As may be expected from the previous discussion, lesions of the CeN, but not the BLA, impair the capacity of an aversive CS to suppress instrumental responding (Killcross et al., 1997b).

#### The CeN is required for conditioned direction

Lesions of the CeN impair appetitive conditioned approach or “autoshaping” (Parkinson et al., 2000; Cardinal et al., 2002b; though see Chang et al., 2012a) by preventing animals from acquiring an approach response, namely by preventing animals from learning to approach either CS, while BLA lesions impair discriminative approach as described above. Furthermore, reduced excitatory glutamatergic transmission in the CeN also impairs conditioned approach, as assessed in *gria2* knockout mice, which express fewer GluR2-containing AMPA receptors in the CeN (Mead and Stephens, 2003a).

The CeN is also required for aversive conditioned direction (avoidance), which can be assessed using a Sidman active avoidance procedure. In this task, rats are trained to shuttle between two chambers to avoid footshock, leading to competition between the avoidance response (shuttling) and conditioned freezing. In a subpopulation of rats that spent much of the time at test freezing, lesions of the CeN rescued these “poor avoiders” and allowed them to shuttle between chambers to avoid the footshock, suggesting that they had learned the instrumental avoidance contingency, but were unable to express the avoidance response due to competition between engaging in freezing behavior and shuttling (Choi et al., 2010; Lázaro-Muñoz et al., 2010). CeN lesions may therefore either have attenuated the general motivational properties of the US, or prevented the habitual freezing response to the US such that subjects no longer engaged in freezing behavior, and were able to perform the instrumental avoidance response governed by the sensory-specific features of the US (Killcross et al., 1997b; Lázaro-Muñoz et al., 2010).

## Amygdala, striatal and prefrontal circuits supporting appetitive and aversive behaviors

**Figure 1:**
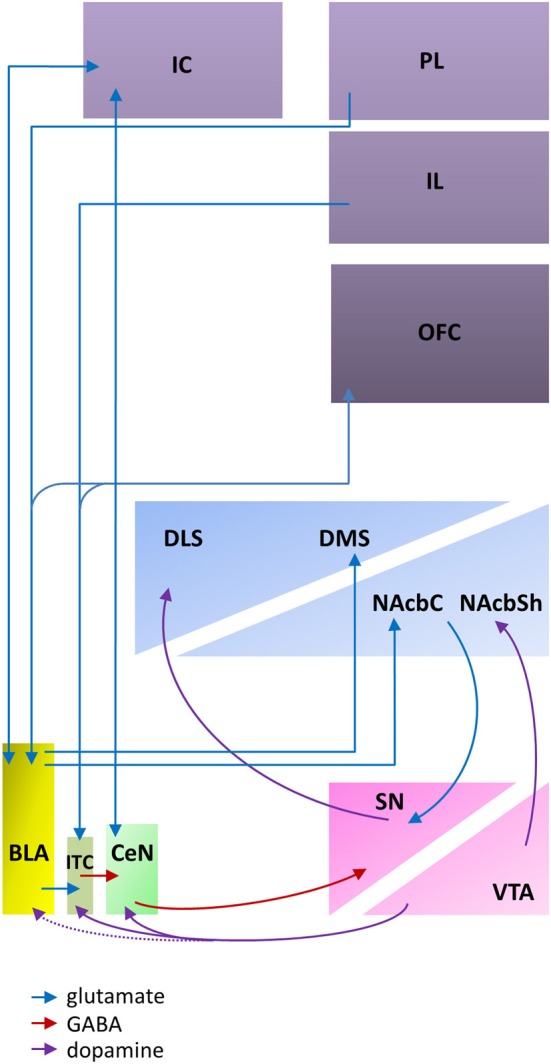
**An abbreviated diagram of amygdala connectivity with the cortex and striatum; refer to text for details on the functions of these connections**. Abbreviations: BLA, basolateral amygdala; CeN, central nucleus of the amygdala; DLS, dorsolateral striatum; DMS, dorsomedial striatum; IC, insular cortex; IL, infralimbic cortex; ITC, intercalated cells; NAcbC, nucleus accumbens core; NAcbSh, nucleus accumbens shell; OFC, orbitofrontal cortex; PL, prelimbic cortex; SN, substantia nigra; VTA, ventral tegmental area. The dashed line indicates weak connectivity.

The evidence reviewed above indicates that the BLA and CeN are necessary for the psychological processes by which pavlovian CSs influence instrumental behavior, including conditioned direction, conditioned motivation and conditioned reinforcement. However, the amygdala does not by itself support the complex behaviors supported by these psychological processes; instead, the amygdala constitutes part of a wider network within the corticostriatal circuitry (Figure 1).

### BLA projections to the striatum support CS control over instrumental behavior

The BLA projects to both the ventral and medial striatum, including the nucleus accumbens (NAcb) and the dorsomedial striatum (DMS). These glutamatergic projections (Kelley et al., 1982) to both the NAcb and the DMS converge with dopaminergic inputs from the ventral tegmental area (VTA; Floresco et al., 2001; Floresco, 2007). This convergence has been shown to be a requirement for reward-seeking behaviors (Ambroggi et al., 2008) and fearful behaviors (Fadok et al., 2010) that are guided by pavlovian CSs. Furthermore, through reciprocal dopaminergic projections connecting the midbrain to the dorsal striatum (Haber et al., 2000; Belin et al., 2009), the BLA can influence motor output regions of the dorsal striatum.

### BLA connections to the PFC support integration of the affective value of CSs with current motivational state

The BLA has extensive reciprocal projections with the PFC, including afferents to both the prelimbic (PL) and infralimbic (IL) cortices (Krettek and Price, 1977). However, the projections from the PL and IL have distinct effects on amygdala activity and may provide a potential mechanism for balancing the impact of an explicit CS-US association stored in the BLA and the more general excitation evoked by a stimulus maintained by CeN circuitry. Excitatory inputs from the PL project directly onto the BLA (Cassell and Wright, 1986; Vertes, 2004), enhancing BLA activity and BLA-mediated inhibition of CeN outputs (Burgos-Robles et al., 2009) whereas excitatory inputs from the IL to intercalated cells (ITC) between the BLA and CeN (Cassell and Wright, 1986) disinhibit CeN activity modulated by the BLA (Quirk et al., 2003; Li et al., 2011a).

The orbitofrontal cortex (OFC) modulates amygdala activity in terms of current motivational states and goal assessment via its extensive reciprocal connectivity with the BLA (Leichnetz and Astruc, 1975; McDonald, 1991) along with projections to the ITC that are thought to maintain a homeostatic modulation of input to the CeN (Ghashghaei and Barbas, 2002). Functionally, this connectivity between the OFC and the BLA is required for the updating of CS representations; for example, reversal learning (Schoenbaum et al., 2003; Stalnaker et al., 2007) or sensitivity to reinforcer devaluation (Baxter et al., 2000), whether through procedures such as “specific satiety,” in which a reinforcer is devalued immediately before behavioral testing by allowing the animal to consume it *ad libitum* (Balleine et al., 2003) or through direct devaluation of an oral reinforcer by induction of gastric malaise (Pickens et al., 2003). A more general view suggests that BLA-OFC connectivity is required for new information to influence behavior (Schoenbaum and Esber, 2010).

This has led to the suggestion that BLA neurons, and neurons in the OFC, represent the overall value of the animal's state (Belova et al., 2008). Interestingly, these value coding neurons are not anatomically segregated, but form distinct circuits which are dynamically modulated as the animals use CSs to predict rewarding and aversive outcomes (Herry et al., 2008; Zhang et al., 2013). How these functionally separate but anatomically indistinct populations of neurons interact with the rest of the corticostriatal circuitry requires further investigation.

### The CeN is part of a circuit by which pavlovian CSs generally activate behavior and support behavior based on S-R associations

As for the BLA, the CeN receives highly processed sensory inputs directly from the thalamus and the cortex (Turner and Herkenham, 1991; McDonald, 1998) in addition to receiving input from the BLA complex via the ITC (Royer et al., 1999; Fudge and Emiliano, 2003) that are modulated by PFC inputs (McDonald et al., 1996; Fisk and Wyss, 1999). The ITC provide a feed-forward network allowing not only excitatory cortical regulation of BLA input onto the CeN (Royer et al., 1999), but also dopaminergic reduction of cortical influence on the ITC (Marowsky et al., 2005). This is provided by the midbrain dopaminergic system that originates in the ventral lateral tegmentum, and traverses the VTA and substantia nigra (SN) with preferential connectivity to the ITC and CeN (Ungerstedt, 1971; Swanson, 1982; Deutch et al., 1988) potentiating the learning of pavlovian CSs (Harmer and Phillips, 1999). Importantly, though the activity of the CeN can be modulated by that of the BLA, the CeN receives and makes projections of its own; it is not simply an output structure for the BLA (Cardinal et al., 2002a; Balleine and Killcross, 2006).

By contrast to the projections of the BLA, the CeN projects to the brainstem and the midbrain, including areas such as the substantia nigra pars compacta (SNc; Lee et al., 2010), the periaqueductal gray (Rizvi et al., 1991), and the hypothalamus (Gray et al., 1989). These connections together are consistent with a role in mediating motor, autonomic, reflexive, and neuroendocrine responses to a pavlovian CS. Though there are no direct projections from the CeN to the striatum (McDonald, 1991), the impact of CeN activity on invigorating more complex behaviors of approach or avoidance (McDannald et al., 2004; Corbit and Balleine, 2005) may be through indirect connectivity with the striatum. Retrograde labeling studies show heavy projections from the medial CeN to the SNc and only limited projections to the VTA (Lee et al., 2010). These CeN projections are primarily targeting the lateral third of the posterior SNc (Zahm et al., 1999; Fudge and Emiliano, 2003), an area with direct dopaminergic connectivity to the dorsolateral striatum (DLS) and only minimal influence on the ventral striatum (Gerfen et al., 1987; Prensa and Parent, 2001). Therefore, in addition to the connections to the DLS via the BLA-NAcbC pathway recruiting reciprocal dopaminergic loops across the midbrain (Haber et al., 2000; Belin et al., 2009), an indirect pathway that utilizes the CeN to SNc to DLS circuitry (Belin et al., 2013) may also influence motor behavior, generally invigorating CS-induced behavior without discrimination between CSs.

### At the systems-level, there is no distinction between appetitive and aversive associations

The amygdala has the appropriate anatomical and neurochemical connections with the striatum and cortex to support its function of storing CS-US associations and modulating reward, reinforcement, and motor processing, independent of the valence of the reinforcer (Figure 1). The BLA and CeN differ in their connectivity in a number of ways that reflect the different functions hypothesized in the parallel processing model: they have different afferent and efferent projections (Pitkänen et al., 1997) though both areas receive highly processed sensory inputs from the cortex and the thalamus (Turner and Herkenham, 1991; Li et al., 1996). Both areas project to the striatum, though the CeN projections are indirect; and both areas have reciprocal innervation with different regions of the prefrontal cortex (PFC). The anatomical circuitry does not distinguish between information about aversive or appetitive CSs; furthermore, evidence from neuronal recordings suggests that highly overlapping populations of neurons encode aversive and appetitive CSs within the amygdala itself.

The “value-coding” neurons found in the amygdala (Belova et al., 2008) are consistent with the view that the BLA and CeN represent sensory-specific and generalized affective representations, respectively. Zhang et al. (2013) observed different responses to CSs and USs in different regions of the amygdala; visual responses to a fixation point in a reversal task and to CSs resulted in more prevalent and faster neuronal responses in the lateral regions of the amygdala, while responses to liquid reward and air puff (multimodal USs) were more evenly distributed through the medial-lateral extent of the amygdala (most likely representing the CeN). Although not completely defined, these results are consistent with the parallel model of amygdala processing (Cardinal et al., 2002a; Everitt et al., 2003; Balleine and Killcross, 2006).

## Interactions between the aversive and appetitive systems

As considered above, the requirement for the BLA and CeN in supporting the processes that underlie conditioned reinforcement, conditioned motivation and conditioned direction appears to be independent of the valence of the reinforcer. It is therefore worth considering whether there is any evidence for separate appetitive and aversive systems within the brain. This question can be approached at multiple levels of analysis, including the psychological, systems and neuronal levels.

### Psychological theories of appetitive and aversive interactions

As previously discussed, the amygdala can represent appetitive and aversive CSs, however, it is difficult to convert an excitor of one motivational system (e.g. a signal that predicts an aversive footshock) into an excitor of the opposite motivational system (e.g. a signal that predicts food reward; Dickinson and Pearce, 1977; Dickinson and Dearing, 1979). According to traditional psychological models, this is due to the inhibitory interactions hypothesized to exist between the appetitive and aversive motivational systems in the brain (Konorski, 1964, 1967). These inhibitory interactions not only allow appetitive and aversive CSs to produce either appetitive or aversive motivational responses, respectively, they also enable another class of stimuli, *conditioned inhibitors*, to produce equivalent behavior to that seen with the presentation of an excitatory CS of the opposite motivational system. For example, a conditioned inhibitor of fear, referred to as a “*safety signal*” (Christianson et al., 2012) signals the absence of an aversive event and in doing so inhibits the aversive motivational system; this hypothetically leads to a disinhibition of the appetitive system so that appetitive behavior can be engaged during the safety signal. Thus, studies of conditioned inhibition emphasize the need to consider both motivational systems when attempting to understand how inhibitory associations are processed and highlight the limitations of considering stimuli to be solely within either an appetitive or an aversive domain.

In addition to acting as inhibitors of the aversive motivational system, safety signals may also possess appetitive qualities. The effects of safety signals on instrumental avoidance behavior have suggested that safety signals can act as positive reinforcers (Rescorla and Lolordo, 1965; Weisman and Litner, 1969; Dinsmoor and Sears, 1973; Morris, 1975) indicating that a conditioned inhibitor of fear has appetitive conditioned value. In accordance with its hypothesized function in storing CS-US associations, the amygdala may be a key area in representing the affective value of safety signals. Learning about safety signals induces a long-lasting synaptic depression of fear CS-evoked activity in the lateral amygdala that correlates with behavioral fear reduction (Rogan et al., 2005). However, changes in the spine size of amygdala neurons, indicative of synaptic plasticity and learning, indicate both increases and decreases following fear conditioning and safety conditioning, respectively (Ostroff et al., 2010), suggesting that the BLA is involved in the processing of both conditioned fear excitors and conditioned fear inhibitors—associating CSs with specific motivational outcomes independent of CS valence.

### BLA neuronal responses to appetitive and aversive stimuli

The BLA is necessary for the control of behavior by specific CSs and enables individuals to use sensory-specific outcomes to guide both instrumental behavior (Balleine et al., 2003; Yun and Fields, 2003) and consummatory behavior, as in the phenomenon of CS-potentiated feeding (Holland and Gallagher, 2003). Electrophysiological studies of amygdala neurons, like the lesion evidence, support the view that the amygdala acts as a CS-US memory storage device within the limbic corticostriatal circuitry. Early studies using single-unit recordings revealed neurons in the amygdala that were activated during motivated behaviors such as eating, drinking and sex (Rolls, 1972). More generally, electrophysiological studies support the hypothesis that the BLA encodes the sensory-specific association of a CS with its affective outcome; the firing of BLA neurons correlates with the coding of the reinforcing and motivational properties of CSs (Tye and Janak, 2007) and, as demonstrated for discriminative fear conditioning, this encoding can be CS-specific (Collins and Paré, 2000). Though correlative, this suggests that neurons within the amygdala can encode “affective value.” “Value-coding” neurons can be operationally defined as neurons that preferentially respond to rewarding or aversive CSs as animals exhibit approach and avoidance behaviors, respectively. Defined in this manner, value-coding neurons have been identified in the primate amygdala using tasks in which reinforcement contingencies were reversed with “positive” and “negative” neurons seen to track value consistently across different sensory events (Paton et al., 2006; Belova et al., 2007; Morrison and Salzman, 2011; Morrison et al., 2011; Barberini et al., 2012; Zhang et al., 2013).

Though there are populations of neurons that respond primarily to appetitive or aversive stimuli (Paton et al., 2006; Belova et al., 2007; Tye and Janak, 2007), single-unit recordings made in the rat BLA suggest that the encoding of appetitive and aversive stimuli can be highly similar. Using a procedure that allowed the parallel investigation of fear, reward, and safety learning in the same animals, Sangha et al. (2013) found different populations of neurons responsive to fear-associated CSs and safety signals, as well as neurons responsive to both safety signals and appetitive CSs. Although behaviorally the rats were able to distinguish the differing cues in this study, there was considerable overlap in neuronal activity, with subsets of neurons responding to all three cues, none of the cues, or only to the fear and reward cue. Rather than necessarily providing evidence for a common appetitive system mediating fear-inhibiting stimuli and rewarding stimuli, this study highlights the complicated nature of circuits within the amygdala that process not only appetitive and aversive associations, but also *stimulus saliency* and *prediction error*. The balance of this processing, and its subsequent effects on behavior mediated by the corticostriatal circuitry, requires further investigation.

## Beyond pleasure and pain: encoding of stimulus salience and reinforcer expectation in the amygdala

The amygdala does not, however, only encode appetitive or aversive associations. Following pavlovian conditioning, some primate amygdala neurons modulate their responses to rewards and aversive stimuli, acting as value coding neurons guiding either appetitively or aversively motivated behavior (Zhang et al., 2013). In other cells, however, effects of expectation on responses were similar for both rewards and punishment, suggesting that some neurons are involved in more arousal-specific responses, reflecting the intensity of activation in the motivational systems rather than the specific valence of a reinforcer. Both valence-specific and arousal-specific neurons in the amygdala were found to be modulated by expectation of reward; initially high levels of firing in these neurons decreased as monkeys learned the CS-US associations, akin to a reduction in a prediction error signal, which aids reinforcement learning through comparison of expected and received reinforcement. Theoretically, the *Pearce-Hall model* predicts that events will be better attended to, and hence learned about faster, when their consequences are surprising or unexpected (Pearce and Hall, 1980). This is similar to empirical observations in the rat BLA, where responses to reward are also consistent with prediction error signals that follow the Pearce-Hall model, as the same neurons fired whether the reward unexpectedly increased or decreased in value (Roesch et al., 2010). These neuronal responses in the BLA differ to those measured in the striatum, which more closely approximate the *Rescorla–Wagner model* (Rescorla and Wagner, 1972) in that the activity of neurons was higher when reward was better than expected, but showed suppression when reward was worse than expected (Delgado et al., 2003; O'doherty et al., 2003; Li et al., 2011b). Neurons within the CeN, however, have only been shown to fire specifically to the omission of an expected reward, and no more to the surprising delivery of a reward than to an expected reward (Calu et al., 2010). This is consistent with lesion studies showing that the CeN is critical for allocating attention for increased processing of events after downshifts but not upshifts in reward value (Holland and Gallagher, 1993). The contrasting responses of the BLA and the CeN support the parallel processing model of amygdala function, with neuronal activity in the CeN mirroring the time course and activity of midbrain dopamine neurons. It has been proposed that negative prediction errors signaled by midbrain dopamine neurons may be conveyed to the CeN, which might then activate basal forebrain cholinergic neurons and other attention-related systems for increases in attention after omission of expected events (Calu et al., 2010). Further characterization of the firing of neurons fitted to prediction error learning models within the BLA and CeN, and in structures projecting to and from these amygdala nuclei, may reveal how these two subnuclei process different representations of a CS-US association.

## Conclusions

We and others (Killcross et al., 1997b; Cardinal et al., 2002a; Balleine and Killcross, 2006) have argued that the amygdala can be conceptualized as a CS-US memory storage device, storing associations between the motivational and affective value of pavlovian CSs, with the CeN representing the general affective properties of pavlovian CSs, and the BLA representing the sensory-specific properties of the same CS. These different functions of amygdala subdivisions lead to differential involvement in specific behaviors produced by dissociable psychological processes such as conditioned reinforcement, conditioned motivation and conditioned direction. These behaviors are not mediated only by the amygdala, but rather by a complex network involving numerous interactions with the corticostriatal circuitry. Convincing evidence suggests amongst other aspects of associative learning, appetitive-aversive associations are coded at the neuronal level rather than at the substrate level, which is seen not only in the amygdala but also in the OFC (Morrison and Salzman, 2011; Morrison et al., 2011), the striatum (Hikida et al., 2010), the lateral habenula (Matsumoto and Hikosaka, 2007) and the VTA (Kim et al., 2012) suggesting that there is little difference in the processing of appetitive and aversive memories throughout the corticostriatal system. Considering the brain in terms of the psychological constructs and processes encoded and supported by different areas, rather than simply appetitive or aversive associations, may prove more productive in understanding the interactions of the “limbic” corticostriatal circuitry, which allows individuals to both secure pleasure and to avoid pain.

### Conflict of interest statement

The authors declare that the research was conducted in the absence of any commercial or financial relationships that could be construed as a potential conflict of interest.
